# Supplementary material for: VGEA: an RNA viral assembly toolkit
Source: PeerJ. 2021 Sep 6;9:e12129. doi: 10.7717/peerj.12129 (PMC8428259; doi:10.7717/peerj.12129)
Supplement: Supplemental Information 2 [file peerj-09-12129-s002.zip › Supplementary_File_2/Performance_Comparison_Assembly_Pipelines_Table_2/CV29/vgea/quast_results/results_2021_05_13_22_59_11/basic_stats/CV29_contigs_GC_content_plot.pdf]

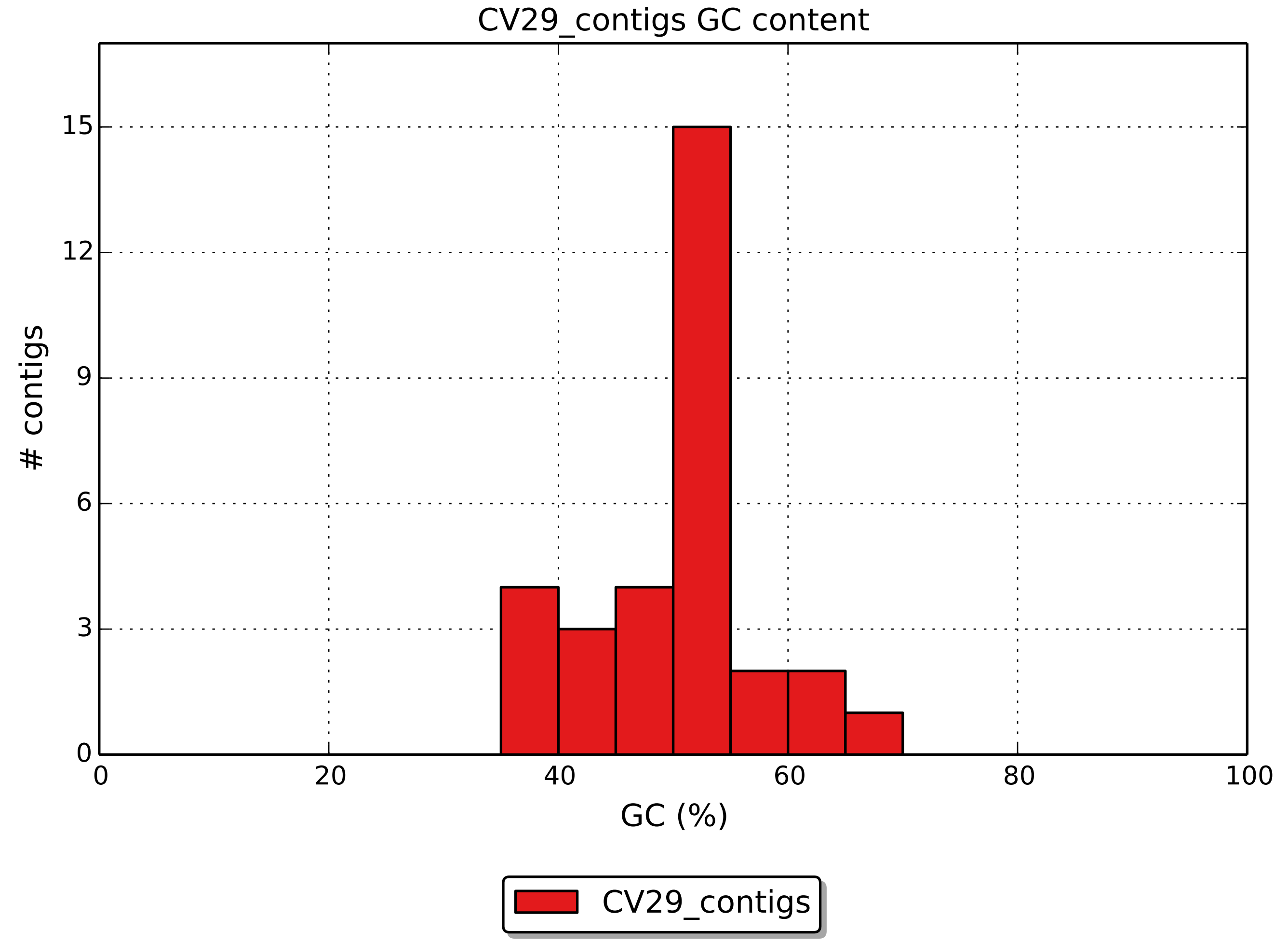
CV29_contigs GC content
15
12
9
6
3
0
contigs
0
20
40
60
80
100
GC (%)
CV29_contigs
